# Ash aggregation enhanced by deposition and redistribution of salt on the surface of volcanic ash in eruption plumes

**DOI:** 10.1038/srep45762

**Published:** 2017-03-31

**Authors:** Sebastian B. Mueller, Paul M. Ayris, Fabian B. Wadsworth, Ulrich Kueppers, Ana S. Casas, Pierre Delmelle, Jacopo Taddeucci, Michael Jacob, Donald B. Dingwell

**Affiliations:** 1 Ludwig-Maximilians-Universität (LMU) München, Earth and Environmental Sciences, Munich, Germany; 2 Earth and Life Institute, Université Catholique de Louvain, Louvain la Neuve, Belgium; 3 Istituto Nazionale di Geofisica e Vulcanologia, Rome, Italy; 4 Glatt Ingenieurtechnik GmbH, Verfahrenstechnik, Weimar, Germany

## Abstract

Interactions with volcanic gases in eruption plumes produce soluble salt deposits on the surface of volcanic ash. While it has been postulated that saturation-driven precipitation of salts following the dissolution of ash surfaces by condensed acidic liquids is a primary mechanism of salt formation during an eruption, it is only recently that this mechanism has been subjected to detailed study. Here we spray water and HCl droplets into a suspension of salt-doped synthetic glass or volcanic ash particles, and produce aggregates. Deposition of acidic liquid droplets on ash particles promotes dissolution of existing salts and leaches cations from the underlying material surface. The flow of liquid, due to capillary forces, will be directed to particle-particle contact points where subsequent precipitation of salts will cement the aggregate. Our data suggest that volcanically-relevant loads of surface salts can be produced by acid condensation in eruptive settings. Several minor and trace elements mobilised by surface dissolution are biologically relevant; geographic areas with aggregation-mediated ash fallout could be “hotspots” for the post-deposition release of these elements. The role of liquids in re-distributing surface salts and cementing ash aggregates also offers further insight into the mechanisms which preserve well-structured aggregates in some ash deposits.

The fast release of over fifty different major, minor and trace elements from volcanic ash on contact with water has historically been attributed to the presence of soluble surface salts^1^. These salts have environmental relevance in terrestrial and aquatic systems, and may alter the chemical reactivity of volcanic ash in the atmosphere^2^. Soluble salts may also play an important role in the cementation of ash aggregates^3^,^4^, and thus may influence ash sedimentation rates, ash dispersal, and deposit properties. Investigating the various mechanisms of gas-ash interactions which emplace soluble salts therefore offers insight into the physical (e.g., deposit thickness distributions) and chemical effects of ash on the natural and human environments. In the last decade, several studies have investigated the formation of salts via adsorption of volcanic gases (SO_2_, HCl, HF) on ash surfaces at temperatures ranging from magmatic to atmospheric^5^,^6^,^7^. However, recent numerical studies^8^ have also examined other mechanisms of salt formation, such as the condensation of acidic liquid droplets onto ash surfaces^9^. During transport in eruption plumes and pyroclastic density currents at elevated temperature and cold volcanic clouds at ambient temperature, the surface of volcanic ash particles may become partially or fully coated with liquid droplets^10^,^11^. These liquid droplets can be formed by condensation of volcanogenic acid solutions of HCl, HF or H_2_SO_4_^7^,^9^, and can be highly acidic; in simulations^12^ using the Active Tracer High Resolution Atmospheric Model (ATHAM), HCl dissolved into suspended water droplets achieved concentrations of 0.1–10 M during the first hour after an eruption. Acidic liquid droplets condensing onto the ash surface will dissolve its glass and mineral constituents, as well as any pre-existing soluble salts^8^. Upon subsequent evaporation, sulphate and halide salts may become saturated in the condensed liquid and precipitate or re-precipitate. Because suspended ash particles traverse an array of temperature, humidity and chemical regimes, the chemistry and volume of the liquid film, and consequently, the solubility of different surface deposits, may fluctuate. Accordingly, salt assemblages on post-depositional ash surfaces may be the product of repeated cycles of dissolution and precipitation.

The variables and processes which govern the formation and evolution of liquid droplets and coatings on ash surfaces are difficult to monitor *in-situ*, and reconstruction of syn- or post-eruptive processing of ash surfaces cannot be easily obtained from post-depositional characterisation. While characterisation of ash surfaces^13^,^14^ or analysis of spatial variations in leachate chemistry and ash deposit properties^15^ offer detailed insights, targeted experimental work is necessary for the discrimination of possible mechanisms. Here, we investigate the process of liquid film development and salt formation and re-precipitation on ash surfaces directly, using dispersed aqueous solutions in fluidised particle mass comprised of salt-doped natural volcanic ash and synthetic glass bead materials. These findings confirm that acid condensation-driven surface dissolution is a key mechanism for rapid, large salt formation which has a clear relevance to ash aggregation and both in-plume and atmospheric processing of ash surfaces. Our results offer insight into the conditions which may dictate volcanic ash deposition and their chemical impacts.

## Materials and Methods

We use two granular materials with particle diameters of <90 μm; (1) synthetic spherical soda-lime silicate glass beads from *Kremer Pigmente* as a synthetic analogue material with well-constrained chemistry, density and surface area, and (2) phonolitic ash as a natural analogue with irregular surface roughness and intra-particle porosity, quarried from pyroclastic deposits from the lower unit of the 13 ka Laacher See eruption (East Eifel volcanic field, Germany^16^, see Table 1).

The particles were loaded in a fluidised bed system (ProCell^®^ Lab System, *Glatt Ingenieurtechnik GmbH*) (1) initially to dope the surfaces with soluble salt (NaCl), and (2) subsequently to expose them to liquid (de-ionized H_2_O or HCl) in turbulent spraying (see also Data Repository and Mueller *et al*.^4^ for fluidized bed design). The ProCell^®^ Lab suspends a granular particle mixture within an upwardly directed gas stream under controlled temperature, bulk turbulence and humidity conditions, generating a fluidised bed when the drag forces exerted by the gas stream exceed the weight of the particles^17^. Through a nozzle, liquid droplets (at controlled composition, size and temperature) have been added to the fluidised bed and deposited on the particle surfaces.

**Figure 1:**
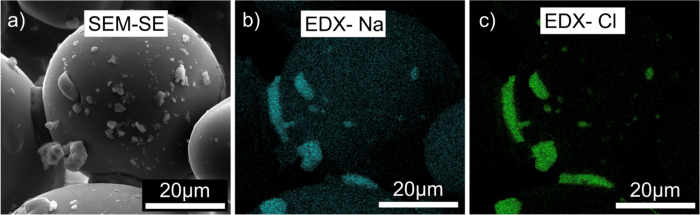
EDX mapping of soda-lime glass bead aggregates. (**a**) Shows a secondary electron (SE) image of the mapping area. (**b**) Highlights Na and (**c**) Cl content which, in combination with a), can be identified as NaCl crystals sitting on glass bead surfaces and in connection points of glass beads as a cementing agent.

In step (1) we sprayed variably concentrated NaCl-H_2_O brine at a rate of 7 ml min^−1^ at 50 °C using a nozzle pressure of 100 kPa. Thereby, we coated the particles with droplets or a film from which, upon evaporation, salt crystals precipitated. The salts were identified as halite (NaCl) crystals using energy dispersive x-ray (EDX) spot analysis and mapping associated with scanning electron microscopy (SEM, Fig. 1). SEM analysis was performed at the HP-HT lab of *INGV Roma*. Initial doping was performed to provide a salt source for remobilization in step (2) because in natural plume environments rapid salt formation after fragmentation is common before a more protracted trajectory down-plume^18^. Effective halite loads were determined from conductivity measurements with an inoLab Cond 730^®^, manufactured by *Wissenschaftliche Technische Werkstätten GmbH*, Germany (calibrated using H_2_O-NaCl solutions of known concentration) and were found to be 19–248 and 31–329 mmol kg^−1^ for the glass bead and volcanic ash materials, respectively (Supplementary Table 1). These halite loads are higher than the average median reported from natural ash samples (see Supplementary Material); this granted the artificial aggregates a higher preservation rate and made aggregate production much more time-efficient. However, it was shown that artificial ash aggregation is also possible with lower halite loads such as reported in the global median of ash leachates^4^.

During step (2), we sprayed 250 ml of de-ionized H_2_O or 12 M HCl solution (at a rate of 40 ml min^−1^) into a fluidised bed of glass bead or volcanic ash samples. Also during step (2), in an additional experiment, we sprayed the volcanic ash materials doped with 251 ± 13 mmol kg^−1^ of halite with 250 ml of 0–12 M HCl solution at a rate of 40 ml min^−1^. In all experiments, nozzle pressure was 50 kPa to allow μm-sized droplets to be produced, leading to the formation of liquid coatings on solid particles. The inlet air flux was set to 0.01–0.02 m^3^ s^−1^, depending on particle size and type of material used, and the average process chamber temperature of 40 ± 15 °C, depending on the applied spray rate. Aggregation of particles was observed after no more than 10 seconds for both experiment types.

We conducted all subsequent analysis on experimentally generated aggregates. Aqueous leaching was used to characterise pre- and post-experiment surface salt loading. Aggregates were immersed in deionised water for one hour at a solid-solution mass ratio of 1:250, and the concentrations of Al, Ca, Cl, Fe, K, Mg, Mn and Si in solution measured following analytical protocols^5^. However, Fe data were subsequently discarded as the possibility of Fe contamination from the corrosion of the stainless-steel tank walls could not be discounted. The granulometry of selected samples was determined using a *Coulter LS-230* laser diffraction particle size analyser (Fraunhofer optical model, imaginary/real refractive indices of 0.001/1.52 for glass beads and 0.1/1.52 for volcanic ash). The specific surface area measurements were made on selected samples by application of the Brunauer-Emmett-Teller (BET) theory to argon adsorption measurements conducted at −196 °C using a *Micrometrics Gemini 1303* surface area analyser. Selected aggregates were analysed by FEG-SEM using a *JEOL JSM 6500* field emission scanning electron microscope to investigate the morphology and chemistry of the surface deposits formed during the experiments.

## Results

**Figure 2:**
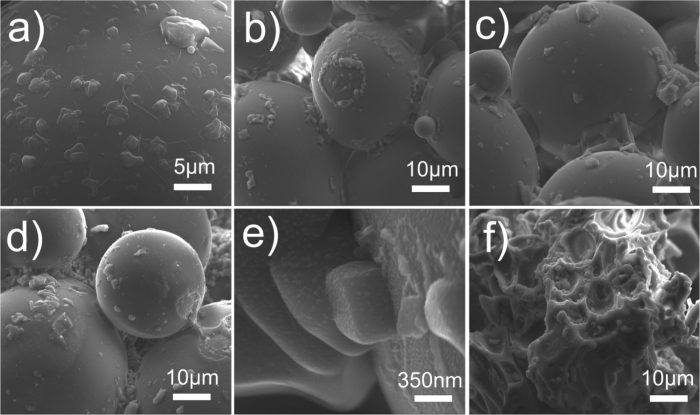
SEM images of experimental particles after (**a**) halite doped glass bead materials produced in experimental step 1, (**b**) halite doped glass bead materials after spraying with deionised water, (**c**) un-doped glass bead materials after spraying with 12 M HCl, (**d**) halite doped glass bead materials after spraying with 12 M HCl, (**e**) surface nodules on halite doped glass bead materials after spraying with 12 M HCl, and (**f**) halite doped volcanic ash materials after spraying with 12 M HCl.

When exposed to HCl solutions of varying concentration, volcanic ash and synthetic glass bead particles undergo dissolution of (1) pre-existing surface salts and (2) the underlying surface. The initial halite doping process produced discrete crystals or clusters of crystals which are evenly dispersed across the material surfaces (Fig. 2a). However, after spraying during turbulent experiments, the distribution and morphologies of surface halite are modified from those produced by the initial doping. When water is used as the sprayed liquid phase, we show that a combination of discrete euhedral and subhedral halite crystals (see Data Repository) are preserved on the glass bead surfaces (Fig. 2b), some of which are several 10 s μm in size. These are clustered around particle-particle contact points. If a particle broke off after the cementation process, isolated ring structures were left behind, indicating the former position of an adhering particle. When 12 M HCl is used as the liquid phase for glass bead materials both without (Fig. 2c) and with (Fig. 2d) a pre-existing halite load, we observe similar structures in addition to extensive patchy regions of sub-micron sized nodules on the surface of ash particles and mega-crystals of halite (Fig. 2e). When volcanic ash is used, the smaller starting grain size distribution (see Supplementary Information) and more complex and irregular morphology precludes the formation of large halite assemblages, and instead, smaller (<5 μm) halite deposits and sub-micron nodules cluster in cavities, cracks and other favourable topographic features (Fig. 2f). These features were observed in all volcanic ash samples analysed, irrespective of the concentration of the applied HCl solution.

For the glass bead materials, analytical leachates are dominated by Na and Cl (60–139 mmol kg^−1^) with minor quantities (1–6 mmol kg^−1^) of Ca and Si, while all other elements were present in concentrations below 1 mmol kg^−1^. The ratio of soluble Na in leachate solutions, termed Na_*s*_ (where a subscript *s* denotes a measured soluble cation concentration throughout), to soluble Cl_*s*_ is approximately 1, while the ratio of Na_*s*_ to the sodium content we would predict from the known pre-existing halite load on the initial material, termed Na_*p*_ (where a subscript *p* denotes the predicted soluble concentration), decreases from 3.8 to 0.4 as the pre-doped applied halite load increases. Analytical leachates from the volcanic ash materials after exposure to HCl are similarly dominated by Na and Cl (206–1308 mmol kg^−1^). Unlike the glass bead materials, the ratio of Na_*s*_ to Cl_*s*_ in 12 M HCl experiments is 0.4 ± 0.2, and increases from 0.4 to 0.9 with decreasing HCl concentration from concentrations of 12 M to 0.7 M. The ratio Na_*s*_/Na_*p*_ decreases from 6.6 to 1.9 as the applied halite load increases, and is approximately 1.5 ± 0.5 for the aggregates formed under varying HCl concentrations. This can be attributed to the decreasing proportional significance of Na extracted from the material surface by HCl dissolution, as the magnitude of the pre-existing halite load increases. Further, the differences in the absolute values of the Na_*s*_/Na_*p*_ ratios between the two materials are attributable to the incorporation of particles of particular grain size into the final aggregates. For example, for the glass bead material, Na_*s*_/Na_*p*_ decreases below unity, implying that per unit mass there is less NaCl present in the final aggregates than cumulatively on the surface of loose glass beads. This is possibly attributed to the slightly lower content of fine particles in the aggregates relative to the starting material. The reason for this observation is not clear yet. In contrast, the volcanic ash aggregates show a finer mode than the starting loose ash and exhibit Na_*s*_/Na_*p*_ ratios greater than unity; this is likely attributable to the contribution of Na mobilised by ash surface dissolution to the pre-existing halite load. These findings can also be shown by estimates of geometric specific surface area (SSA_geo_), which were calculated from application of a spherical approximation to the particle size distributions of the aggregate materials and are consistently half that of the initial glass beads (Supplementary Information).

### Liquid spreading and redistribution of salts

**Figure 3:**
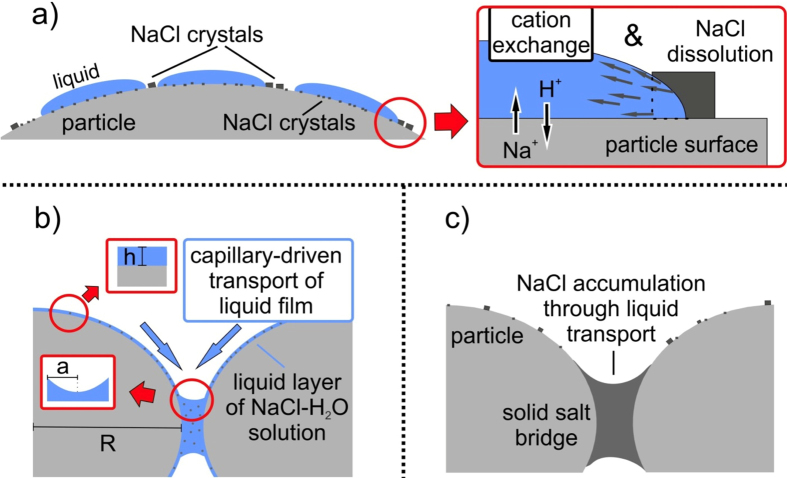
(**a**)Liquid droplets of HCl or H_2_O spread around the particle as a liquid film, dissolving the NaCl coating and triggering cation exchange with the underlying particle surface. (**b**) Capillary forces accumulate NaCl-H_2_O brine at particle-particle contact points, forming liquid bridges. The liquid layer thickness *h*, particle radius *R* and the curvature of radius in the liquid neck *a* are labelled. (**c**) Evaporation of the liquids during drying processes leads to precipitation of a solid NaCl bridge and depletes the particle surface in NaCl crystals.

Comparison between Fig. 2a,b–d demonstrates that the NaCl crystals were redistributed during liquid spraying and aggregation. In the glass bead samples, evenly distributed crystals on the initially doped surfaces became localized NaCl crystal assemblages at particle-particle contact points. NaCl assemblages between coarse glass beads are larger in size (<10 μm) compared to those between fine bead materials (<2 μm; Fig. 2c,d), conveying less aggregate stability to fine materials. We interpret the formation of NaCl assemblages to be due to the capillary-driven action of liquid films toward contact points. The deposition, distribution and motion of thin films and drops on surfaces is a complex problem, however, to explore this, we consider a spherical particle of radius *R* with continuous film thickness *h*. The surface area of the film with air is *s*_*i*_ = 4*π(R* + *h*)^2^. This can be minimized by bunching the liquid in a collar around the contact of two particles^19^. The flow of the film toward the contact area where the curvature of the liquid surface is largest is driven by the excess Laplace pressure *P* = 2*Γ*/*a*, where *a* is the radius of curvature at the contact point. Since *a* is smaller (tighter curvature) than *R* + *h* for the relative radii during liquid collar formation, the flow is toward the contact^20^. Additionally, during drying of the liquid layer and collar, salts precipitate. We use these simple arguments to suggest that during or after dissolution of surface salt load, flow in the liquid film resulted in a liquid collar around the contact point of particles, which in turn led to the precipitation of salt at these collars on drying (Figs 2 and 3). This effectively cemented the aggregates, preserving them for analysis. This process of (1) dissolution of soluble minerals on the surface of ash particles, (2) transport of the resulting liquid by capillary forces to contact points and (3) crystallisation upon evaporation can be rather quick as evidenced from deposits of the 2006 eruption of Tungurahua volcano, Ecuador. In this case, accretionary lapilli were found exclusively close to Chambo river. During the eruption, pyroclastic density currents had temporarily dammed the river. The hot (200–500 °C) deposits had interacted with the water, causing a secondary plume of elutriated ash and locally increased air humidity^21^.

### Surface leaching of materials

The interactions of volcanic ash particles and aggregates with HCl solutions offer insight into those between in-plume ash particles and acidic liquid droplets. In 12 M HCl experiments, there are strong relationships (*r*^2^ > 0.85, when a linear relationship is assumed) between Al_s_, K_s_ and Mn_s_, elements which solely derive from ash surface dissolution; K_*s*_/Al_*s*_ ≈ 0.33, Mn_*s*_/Al_*s*_ = 0.33, Mn_*s*_/K_*s*_ = 0.08. However, no similar relationship is observed between Ca_s_ or Mg_s_ or between these and any of the former elements. This likely reflects the fact that volcanic ash surface constituents do not dissolve at the same rates. Notably, the relative abundance of ratios K_*s*_/Al_*s*_, Mn_*s*_/Al_*s*_ and Mn_*s*_/K_*s*_ are similar to those for the bulk chemistry of the volcanic ash material. Previous studies of volcanic glass (basalt to rhyolite compositions) in low pH environments (pH 4 in dissolution experiments^22^, or pH < 1 in field observations^3^), have shown that metal release rates decrease exponentially with increasing Si content. Silicate dissolution also involves an initially incongruent leaching of univalent and divalent cations from the near surface region; Al release exchange reactions between aqueous H^+^ and Al in the glass structure set in before late-stage dissolution of the Si network takes place^22^,^23^. While at long times of exposure to acidic solutions, the bulk silicate composition should be congruent with the leachate chemistry, over the short exposure times of the current study, it is likely that preferential leaching of alkalis and Al can explain our data.

**Figure 4:**
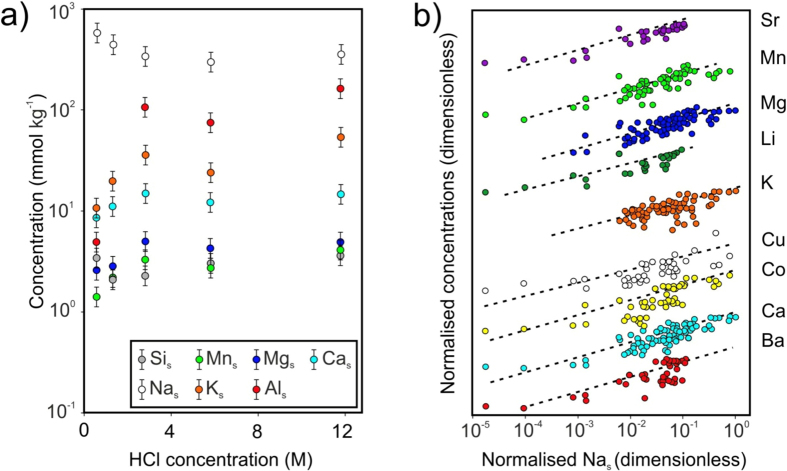
Composite figure displaying (**a**) Na_s_, Al_s_, K_s_, Ca_s_, Mg_s_, Mn_s_, Si_s_ from volcanic ash materials sprayed with varying HCl concentrations; (**b**) selected soluble element concentrations plotted against soluble Na concentrations using data from eleven leachate studies (see Data Repository), normalized to their maxima and offset arbitrarily to be viewable at the same scale, with best-fit power-laws provided to guide the eye and to demonstrate a commonality of slope.

Our observations highlight the influence of both variations in exposure time and in HCl concentration on surface dissolution. In the second experiment series using the volcanic ash material, the decrease in concentrations of soluble Al, Ca, K, Mg and Mn with decreasing HCl concentrations (Fig. 4a) is due to the decreasing leaching efficiency and subsequent dissolution of the silicate network. Additionally, volcanic ash aggregates produced herein and extracted after only 10 seconds of exposure to 12 M HCl solutions produced significant concentrations of soluble elements; on average, concentrations were ~3 times higher than the global median of published ash leachate data^2^. For comparison, at the end of the experiment, after 375 s, concentrations were ~20 times higher. Even considering that the high measured specific surface area (SSA_BET_; 3 m^2^ g^−1^) of the volcanic ash materials amplifies the representation of surface salts in leachate data relative to natural ash samples with lower SSA_BET_ (e.g., 1–2 m^2^ g^−1^)^10^, these results still demonstrate the capacity for salt formation in volcanically relevant quantities on very short timescales.

### Relevance to natural processes

Artificial aggregation experiments with the *ProCell*^*®*^ *Lab* can mimic natural processes. Here we explore the scaling between our experimental setup and natural scenarios. The Reynolds number scales the relative importance of viscous to inertial forces in a complex fluid flow given by


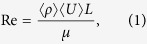


where (for the ProCell^®^ Lab) 〈*ρ*〉 ≈ 0.96 kg.m^−3^ is the mean current density, 〈*U*〉 ≈ 0.09 m.s^−1^ is the mean current velocity, *L* = 0.5 m is a characteristic lengthscale and *μ* = 10^−4^ Pa.s is the suspending fluid viscosity. In the fluidized bed system at the operating conditions used here we find that 10^0^ ≤ Re ≤ 10^3^, compared with an in-plume range 10^7^ ≤ Re ≤ 10^10^ (ref. 24, we anticipate this number to be much lower in more dilute, downwind plume areas) and a range for a pyroclastic density current of 10^6^ ≤ Re ≤ 10^9^ (ref. 25). This suggests our experimental flows are most similar to the more gentle, dilute parts of plumes, which is consistent with (1) the temperatures used here and (2) the fact that we pre-dope the samples with salt loads, which is the case in the down-plume areas of a dispersal axis.

How coupled a particle is to the turbulent motion of the flow is determined by the Stokes number which is given by


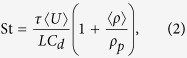


where *τ* is the response time of a particle to turbulent motion^26^, *C*_*d*_ is the particle drag coefficient which is 0.47 and 1.0 for the glass beads and the volcanic ash, respectively, *ρ*_*p*_ is the particle density which is 2,500 and 2,300 kg.m^−3^ for the glass beads and the volcanic ash, respectively. This yields 10^−2^ < St < 10^−1^ for our experiments, implying that the particles are well coupled to the turbulent flow. In plumes this is also the case^24^ and in pyroclastic density currents, the range is 10^−2^ < St < 10^2^ (refs 25, 27), implying the particles straddle the regime divide. This further bolsters our claim that our experiments well replicate the down-plume end member of volcanic ash transport processes.

A viscous Stokes number represents the balance between elastic repulsive forces and a stickability associated with each particle-particle interaction in the plume or experiment and is given by


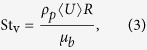


where 0.6 < *μ*_*b*_ < 1.3 mPa.s is the viscosity of the binder fluid film on the particle surface. We estimate that 10^−2^ < St_v_ < 10^0^ in our experiments. A value St_v_ < 1 suggests that viscous attractive forces at impact will result in particle capture and not rebound, which is evidenced by the fact that we produce aggregates at all. Moreover, in nature the presence of aggregates for which a fluid binder was involved suggests that St_v_ < 1 for at least a proportion of particle-particle interactions. In proximal parts of eruption plumes, the rise-speed of particles can be (up to 20 m.s^−1^)^28^ compared to particle velocities in the *ProCell*^*®*^ *lab* (up to 0.2 m.s^−1^), again suggesting that we do not replicate the proximal conditions of turbulence and rather scale to colder, less turbulent down-plume or dilute pyroclastic density current conditions.

We do not scale thermal exchange of heat (Prandtl number) in our system because we use isothermal experiments. In nature, the temperatures of our experiments are indeed found down-plume^29^. Similarly, we do not scale diffusive mass transfer (Peclet or Schmidt numbers) because the pre-doping stage of our experimental protocol implies that the diffusive process that leads to salt formation from the glass chemistry in the first place is complete^18^. This is akin to saying that diffusion is rapid compared with other processes of interest here such as particle-particle interaction processes and aggregation. This is reasonable as the up-plume, hot, proximal area is where the diffusive mass transfer processes will be dominant^18^. In those same regions, however, the turbulence is sufficiently high to impede aggregation. Therefore, the process of diffusive mass transfer and the formation of primary salt loads occurs without competing with aggregation processes. Down-plume, however, the turbulence drops to without our experimental range and the diffusive mass transfer is less operative (lower temperatures).

Binder concentrations reported in this study are higher than a natural average but still within the upper limits of measured ranges. Artificial aggregation with average salt concentrations reported for natural aggregates (~100 s mg kg^−1^) however has been carried out with the *ProCell*^*®*^ *Lab*^4^ with similar aggregation results. Experimental relative air humidity necessary for aggregation (>10%), temperature range (40–60 °C) and particle size distributions (<90 μm) are all naturally applicable.

The above scaling arguments allow us to argue that our experimental work well-replicates less-turbulent, colder parts of eruptions plumes and dilute pyroclastic density currents.

### Implications

Our experimental results represent the first laboratory investigation of the chemical interactions between volcanic ash or glass beads and acid liquid droplets at temperature and time conditions relevant to explosive eruptions. Our experiments suggest that interaction with variably concentrated HCl droplets promote the preferential leaching and dissolution of the ash surface and the precipitation of surficial chloride salts. Excluding the pre-existing halite load, these salts were emplaced as submicron-sized, patchily distributed deposits, similar to those observed on natural ash surfaces^13^,^30^. In experiments conducted with 12 M HCl, volcanologically-relevant quantities of salts were formed within 10 s of exposure, while quantities far in excess of those typically observed in volcanic ash leachates were formed after 375 s. In the latter experiment, such prolonged exposure times may only be relevant to the largest explosive eruptions; 3D plume models^31^ indicate that some ash particles in such events may be exposed to temperature of 40–50 °C for similar periods. Dissolution of the ash surface by acid liquid droplets in large explosive eruptions, particularly those which emit a significant quantity of HCl, may therefore produce ash with high loads of biologically-relevant^2^ and readily-soluble minor elements relative to that from smaller eruptions.

However, not all erupted ash particles will encounter liquid droplets of 12 M HCl, and silicate dissolution at lower temperatures than those utilised in our experiments is significantly slower^32^. Accordingly, it is likely that interactions between ash and acidic liquid droplets in both large and small eruptions produce varying abundances of soluble salts, perhaps even from ash particle to particle according to their individual plume trajectories. Notably, comparison of eight pristine ash leachate data sets^33^,^34^,^35^,^36^,^37^,^38^,^39^,^40^ reveals that Ca, Mg, K, and a number of biologically-relevant minor and trace elements (Ba, Co, Cu, Li, Mn, Sr) increase with increasing Na concentrations (Fig. 4b). This should not be interpreted to indicate that Na is a causative factor, but rather that the coincident increase in the abundance of major (Na, K, Mg, Ca) and both minor and trace elements is consistent with increasing ash surface dissolution by liquid acid films both within and across the different studies.

If acidic liquid films drive the formation of soluble salts in most volcanic eruptions, ash deposits with comparatively high yields of biologically-relevant minor elements may coincide with areas of aggregate-driven sedimentation. Notably, in the retrospective analysis of ash leachates from the May 18^th^ eruption of Mount St Helens, it was noted that a region of low Na_s_/Cl_s_ ratios, indicative of the presence of chloride salts other than halite and attributed to ash interaction with HCl-rich hydrometeors, occurred in an area of aggregate fallout^15^. For the same eruption, our observations can also explain the preservation of well-rounded aggregates in the co-ignimbrite ash fall deposits from afternoon flows on May 18^th^. It was suggested that vaporisation of glacier ice or groundwater promoted aggregation of ash^41^. Leaching of ash from blast deposits revealed a high salt load^34^, which would have encouraged preservation of aggregates^4^. While the pre-existing salt load can be derived from several processes such as pre-eruptive alteration by high temperature gases^15^, or the production of an NaCl brine^42^ which deposits NaCl aerosols on ash surfaces^43^,^44^, the condensation of water or acidic droplets onto ash particles would have dissolved the existing salts, re-precipitating them at particle-particle contact points, cementing the aggregates and permitting their preservation within the deposits.

Volcanic ash aggregates bound by NaCl have been previously described at Sakurajima volcano, Japan^3^. The formation of NaCl-laden aggregates in the current study presents further insight into the mechanisms of salt emplacement on ash surfaces and their environmental fate. The spreading of a liquid film around the ash surface may re-deposit the most soluble salts in areas of favourable surface morphology, while less soluble salts are dispersed across larger regions of the surface. Convincingly, previous studies on ash-gas interactions^13^ and on ash deposits of the 2010 Eyjafjallajökull eruption^30^, have documented thin nanoscale halite coatings on some particle surfaces, similar to those in Fig. 2e. The re-distribution of highly soluble salts from across the ash surface into isolated assemblages potentially obscured or hidden by surface topography could also make them difficult to observe without extensive microscopy analyses, and impede their identification by surface sensitive techniques (e.g., XPS^13^), as their proportional footprint on the ash surface will be small.

Ash aggregation can occur by multiple processes, and does not necessarily require salt-formation to bind particles together^3^,^45^,^46^. Alternative possibilities include rapid alteration of ash surfaces in the presence of liquid water producing coatings of amorphous silica^46^ or high temperature sintering by either viscous flow of hot ash surfaces producing necks or diffusive exchange^47^. However, salt redistribution volcanic ash surfaces or surface dissolution-derived salt formation during any of the mechanisms of initial aggregation will increase the stability of aggregates.

## Additional Information

**How to cite this article**: Mueller, S. B. *et al*. Ash aggregation enhanced by deposition and redistribution of salt on the surface of volcanic ash in eruption plumes. *Sci. Rep.* **7**, 45762; doi: 10.1038/srep45762 (2017).

**Publisher's note:** Springer Nature remains neutral with regard to jurisdictional claims in published maps and institutional affiliations.

## Supplementary Material

Supplementary Material

Supplementary Dataset 2

## Figures and Tables

**Table 1:** Bulk chemical composition of the soda-lime glass beads (SLS) and the phonolitic Laacher See ash (PHN) materials.

Bulk chemistry (wt. %)	SLS^a^	PHN^b^
SiO_2_	72	56.2
TiO_2_	—	0.2
Al_2_O_3_	<*0.1*	20.6
Fe_2_O_3_	—	1.5
FeO	—	0.6
MnO		0.30
MgO	<*0.1*	0.15
CaO	9	1.1
Na_2_O	13	9.4
K_2_O	<*0.1*	5.9

^a^Bulk chemistry provided by *Kremer Pigmente* GmbH, Germany.

^b^Bulk chemistry data averaged from lower- and middle- units of Laacher See deposits^16^.
