# Supplementary material for: Ash aggregation enhanced by deposition and redistribution of salt on the surface of volcanic ash in eruption plumes
Source: Sci Rep. 2017 Mar 31;7:45762. doi: 10.1038/srep45762 (PMC5374634; doi:10.1038/srep45762)
Supplement: Supplementary Material [file srep45762-s1.doc]

**Ash aggregation enhanced by deposition and redistribution of salt on the surface of volcAnic ash in eruption plumes**

Sebastian B. Mueller1,Paul M. Ayris1, Fabian B. Wadsworth1, Ulrich Kueppers1,Ana S. Casas1, Pierre Delmelle2, Jacopo Taddeucci3, Michael Jacob4,Donald B. Dingwell1

1 Ludwig-Maximilians-Universität (LMU) München, Earth and Environmental Sciences, Munich, Germany. 2 Earth and Life Institute, Université Catholique de Louvain, Louvain la Neuve, Belgium. 3 Istituto Nazionale di Geofisica e Vulcanologia, Rome, Italy. 4Glatt Ingenieurtechnik GmbH, Verfahrenstechnik, Weimar, Germany.

Corresponding author: sebastian.mueller@min.uni-muenchen.de

**-- Supplementary File --**

**Supplementary Table 1.** Compilation of aggregate specific surface area (m2 g-1) expressed as geometric specific surface area (GEO), and as measured by argon adsorption and application of the BET theory to the post-experiment aggregates (BET); and analytical leaching data for the measured elements (Na, Cl, Al, Ca, K, Mg, Mn, Si; mmol kg-1) for SLS materials following aggregation experiments with variable halite concentrations after spraying with water (SW1) or with 12 M HCl (SA1-6), and for phonolitic Laacher See ash (PHN) materials following aggregation experiments with variable halite concentrations after spraying with water (PW1); with 12 M HCl (PA1-6a); and with variably diluted HCl solutions (PC1-6). The ratios of Nas:Cls and Nas:Nap, described in the main text, are also given where appropriate. Additionally included is the global mean concentration (GM) of the same measured elements in other volcanic ash leachate studies1.

| **Sample**  **Code** | **Halite (mmol kg-1)** | **Acid conc. (M)** | **Specific surface area**  **(m2 g-1)** | | **Mean**  **PSD**  **(µm)** | **Leachate conc.**  **(mmol kg-1­)** | | | | | | | **Elemental**  **ratios** | | |
| --- | --- | --- | --- | --- | --- | --- | --- | --- | --- | --- | --- | --- | --- | --- | --- |
|  |  |  | GEO | BET |  | Na | Cl | Al | Ca | K | Mg | Mn | Si | Nas/Cls | Nas/Nap |
| SLS | 0 | 0 | 0.09 ±0.00 | *3.4* | 60.5 | 1.24 | <0.1 | <0.1 | 0.9 | <0.1 | <0.1 | <0.1 | 0.5 | - |  |
|  |  |  |  |  |  |  |  |  |  |  |  |  |  |  |  |
| SW1 | 254 | 0 | *-* | *-* | *59.3* | *-* | *-* | *-* | *-* | *-* | *-* | *-* | *-* |  |  |
| SA1 | 19 | 12 | 0.05 ±0.00 | *-* | 61.3 | 72.0 | 72.0 | 0.2 | 2.4 | 0.4 | 0.6 | <0.1 | 1.9 | 1.0 | 3.8 |
| SA2 | 28 | 12 | *-* | *-* | 58.2 | 60.8 | 61.9 | 0.1 | 3.1 | 0.4 | 0.6 | 0.1 | 2.2 | 1.0 | 2.2 |
| SA3 | 36 | 12 | 0.06 ±0.00 | *-* | 60.1 | 64.4 | 66.7 | 0.1 | 2.8 | 0.4 | 0.6 | <0.1 | 1.7 | 1.0 | 1.8 |
| SA4 | 84 | 12 | 0.06 ±0.00 | *-* | 55.1 | 103.4 | 104.8 | 0.2 | 3.4 | 0.6 | 0.8 | <0.1 | 2.2 | 1.0 | 1.2 |
| SA5 | 168 | 12 | 0.05 ±0.00 | *-* | 63.2 | 147.1 | 145.1 | 0.0 | 2.6 | 0.4 | 0.5 | <0.1 | 1.8 | 1.0 | 0.9 |
| SA6 | 248 | 12 | 0.05 ±0.00 | *-* | 60.7 | 142.7 | 138.6 | 0.1 | 1.8 | 0.3 | 0.4 | <0.1 | 5.1 | 1.0 | 0.6 |
| , |  |  |  |  |  |  |  |  |  |  |  |  |  |  |  |
|  |  |  |  |  |  |  |  |  |  |  |  |  |  |  |  |
| PHN | 0 | 0 | 0.31 ±0.04 | 2.9 | *59.36* | *-* | *-* | *-* | *-* | *-* | *-* | *-* | *-* | *-* |  |
|  |  |  |  |  |  |  |  |  |  |  |  |  |  |  |  |
| PA1 | 31 | 12 | 0.29 ±0.03 | *~* | - | 206.1 | 724.1 | 125 | 14.5 | 38.6 | 4.8 | 3.5 | 5.7 | 0.3 | 6.6 |
| PA2 | 43 | 12 | - | 6.1 | 54.8 | 239.1 | 896.9 | 173.8 | 13.4 | 55.7 | 4.6 | 4.3 | 4.4 | 0.3 | 5.6 |
| PA3 | 97 | 12 | 0.27 ±0.02 | 4.2 | 47.2 | 227.3 | 567.3 | 77.5 | 13.6 | 25.7 | 4.5 | 2.8 | 2.9 | 0.4 | 2.3 |
| PA4 | 172 | 12 | 0.28 ±0.02 | *-* | - | 320.5 | 969.5 | 167 | 13.5 | 53.3 | 4.6 | 4.2 | 2.6 | 0.3 | 1.9 |
| PA5 | 254 | 12 | 0.32 ±0.00 | 5.6 | 59.3 | 514.3 | 1308.7 | 203.9 | 15.6 | 70.2 | 4.5 | 5.3 | 2.4 | 0.4 | 2.0 |
| PA6 | 329 | 12 | 0.31 ±0.01 | 5.4 | 59.3 | 632.4 | 1273 | 168.8 | 13.5 | 57.5 | 3.9 | 4.4 | 3 | 0.5 | 1.9 |
| PA6a | 329 | 12 | *-* | *-* | - | 366.4 | 438.8 | 15.7 | 8.2 | 13.3 | 3 | 1.5 | 2.7 | 0.8 | 1.1 |
|  |  |  |  |  |  |  |  |  |  |  |  |  |  |  |  |
| PC1 | 254 | 0 | *-* | 2.8 | 50.9 | 184.9 | 177.2 | 0.3 | 2.1 | 4.6 | 0.6 | 0.1 | 2.3 | 1.0 | 0.7 |
| PC2 | 264 | 0.7 | 0.33 ±0.08 | *-* | - | 585.5 | 621.0 | 5.0 | 2.1 | 4.5 | 0.6 | 0.0 | 2.3 | 0.9 | 2.2 |
| PC3 | 264 | 1.5 | 0.29 ±0.04 | *-* | - | 453.2 | 556.7 | 20.4 | 8.7 | 10.9 | 2.6 | 1.4 | 3.4 | 0.8 | 1.7 |
| PC4 | 254 | 2.9 | 0.35 ±0.04 | 2.6 | 79.8 | 346.3 | 749.4 | 76.4 | 11.3 | 19.5 | 2.9 | 2.2 | 2.3 | 0.5 | 1.4 |
| PC5 | 237 | 5.8 | 0.26 ±0.02 | - | - | 303.1 | 628.7 | 106.7 | 12.4 | 24.6 | 4.3 | 2.8 | 3.1 | 0.5 | 1.3 |
| PC6 | 237 | 12 | - | *-* | - | 359.5 | 977.1 | 164.2 | 15.2 | 36.6 | 5.0 | 3.3 | 2.3 | 0.3 | 1.5 |
|  |  |  |  |  |  |  |  |  |  |  |  |  |  |  |  |
| GM | - | - | - | *-* | - | 17.7 | 33.9 | 2.3 | 54 | 1.9 | 14.4 | 0.4 | 1.0 | - | - |

**Supplementary Table 2 (attached .xlsx sheets).** A compilation of leachate data for various cations used to generate Figure 3b, as reported in eleven previous studies of volcanic ash. Included as a separate digital .xlsx file, including the study of origin, denoted by a two letter code also documented in the .xlsx file, the original sample code, and the leachate concentration reported in mmol kg-1. Data were excluded when leachate compositions were i) not reported, ii) incompletely reported, iii) reported as an average of multiple ash samples, iv) not pristine, or v) reported as being below the detection limit of the prior study.

Reference:

1. Ayris, P. M. & Delmelle, P. The immediate environmental effects of tephra emission. *Bull. Volcanol*. **74**, 1905-1936 (2012).
